# Implementation of the college student mental health education course (CSMHEC) in undergraduate medical curriculum: effects and insights

**DOI:** 10.1186/s12909-020-02438-1

**Published:** 2020-12-11

**Authors:** Qinghua Wang, Tianjiao Du

**Affiliations:** 1 English Department, School of Fundamental Sciences, China Medical University, No. 77 Puhe Road, Shenyang North New Area, Shenyang, Liaoning Province People’s Republic of China; 2 Department of Psychology, School of Humanities and Social Sciences, China Medical University, No. 77 Puhe Road, Shenyang North New Area, Shenyang, Liaoning Province People’s Republic of China

**Keywords:** Medical students, Psychological health, Intervention, Mental health education course

## Abstract

**Background:**

Extant literature reveals that medical students suffer from various mental health problems in the process of learning medicine. However, there are few studies evaluating the implementation of a mental health education course in medical curriculum. The current study aimed to test the effectiveness of an 8-week intensive mental health education course, the College Student Mental Health Education Course (CSMHEC), and to gain further insights on how the course could be improved from students’ feedback.

**Methods:**

This is a quasi-experimental study with both quantitative and qualitative analyses. We recruited 374 first year medical students as our subjects with 188 (age = 17.97 ± 0.65 years, 37.2% male) for the experiment group and 186 (age = 18.02 ± 0.63 years, 40.3% male) for the control group. For quantitative analysis, Depression Anxiety Stress Scales-21 (DASS-21), Chinese College Student Academic Burnout Inventory (CCSABI) and Satisfaction With Life Scale (SWLS) were used and a 5-point Likert scale was used to indicate students’ overall satisfaction with CSMHEC. For qualitative analysis, a thematic analysis method was adopted to gain insights from the feedback of medical students.

**Results:**

Medical students in the experiment group saw a significant decline in psychological distress (*p* < 0.001, *d* = 0.31) and academic burnout (*p* < 0.001, *d* = 1.46), while they experienced a significant increase in life satisfaction levels after the intervention (*p* < 0.001, *d* = 0.48). Compared with students in the control group, students in the experiment group had statistically significant lower levels of psychological distress (*p* < 0.05, *d* = 0.23) and academic burnout (*p* < 0.001, *d* = 0.70), but statistically significant higher levels of life satisfaction in the post-test (*p* < 0.01, *d* = 0.31). Most students in the experiment group were satisfied with CSMHEC and themes extracted in the thematic analysis shed light on how the course could be improved.

**Conclusions:**

Implementing a mental health education course like CSMHEC in medical curriculum can be effective in helping medical students improve psychological health. More research needs to be conducted on further refinement and better design of such a course to implement in medical education.

**Supplementary Information:**

The online version contains supplementary material available at 10.1186/s12909-020-02438-1.

## Background

Medical education is known to be a long and painstaking process that requires hard work and continuous efforts. Such a demanding process may bring about various mental health problems like stress, anxiety, depression and burnout. These problems may lead to a set of serious consequences in medical students, such as poor academic performance [1], sleep disturbance [2], alcohol and drug abuse [3, 4], low self-worth [5] and even suicide [6–8]. As a healthy mental state is important for student well-being in general, which is essential for a medical student to succeed finishing study in medical university and become a qualified health professional thereafter, there is an urgent need to implement effective interventions early.

Literature shows that different interventions have been developed to increase medical students’ mental health literacy and help medical students improve psychological well-being. Davies and colleagues [9] designed an evidence-based intervention called Mental Health First Aid (MHFA) eLearning course and conducted a pilot randomized controlled trial (RCT). The results strongly demonstrated the efficacy of the program in raising medical students’ confidence in helping a friend with mental health problems and in reducing medical students’ stigma towards mental illnesses. A systematic review and meta-analysis [10] on the effectiveness of mindfulness training for medical students and students of other health professions included 19 studies covering both randomized and non-randomized controlled trials. It was found that mindfulness-based interventions were effective in reducing stress, anxiety and depression while enhancing mindfulness, self-efficacy and empathy among health professional students. In addition, the research conducted by Gallego-Gómez et al. [11] used an RCT to evaluate an intervention which combined music therapy and Progressive Muscle Relaxation (PMR) to help nursing students alleviate pressure before exams. The results showed that compared with the students in the control group, students in the experiment group had lower stress levels before the Clinical Nursing exam and achieved significantly higher scores in the exam. Also adopting an RCT design, Sahranavard and colleagues [12] tested the effectiveness of cognitive behavioral therapy (CBT) among female medical students in Iran and found that CBT was effective in lowering medical students’ levels of anxiety while increasing their levels of hardiness and self-efficacy.

Although some medical institutions provided psychotherapeutic programs such as counseling and relaxation training to medical students, it is found that the utilization rate of these psychological services was not as high as expected [13]. The reasons behind this trend are probably that some medical students who suffer from mental health problems are ashamed of seeking help and worried that the revelation of their poor mental states may exert detrimental impacts on their social relationship, academic assessment and career prospects [14]. Studies indicated that people were reluctant to use public mental health services due to the social stigma towards psychiatric disorders [15, 16], and medical students and health professionals are no exception. Therefore, medical educators need to find out a feasible and efficacious approach to helping medical students increase mental health literacy and enhance psychological health. Implementing a mental health education course in medical curriculum may be such a promising approach and we conducted the current research to test the effectiveness of this approach and to gain further insights on how the course could be improved from students’ feedback (e.g. students’ satisfaction levels with and opinions about the course).

In September 2019, China Medical University provided a mental health education course called the College Student Mental Health Education Course (CSMHEC) to first-year medical students. As a pilot program, CSMHEC was not given to all first-year medical students, but only to students of some majors concerning medicine (preventive medicine, nursing, medical imaging, biomedical engineering, basic medical science and health service administration). This is because the schedule was tight for first-year students of other majors concerning medicine such as clinical medicine and dentistry. The aims of CSMHEC are to familiarize students with the definition and standard of mental health, help students develop emotion regulation skills, stress coping strategies, environment adaptation abilities, learning potentials and problem-solving abilities. CSMHEC focuses on college students’ psychological characteristics and aims to help students clarify some irrational beliefs and stereotyped views. By taking this course, medical students are expected to acquire knowledge on basic theories and concepts in psychology, cultivate self-care awareness, develop high adaptability and grasp strategies to deal with distress. The current study aimed to examine the effectiveness of implementing CSMHEC in undergraduate medical education and to identify any potential problems with the course design and course delivery from the implementation.

## Methods

### Setting

This study was conducted at China Medical University, a major medical university in Northeast China. In China Medical University, there are three kinds of programs for undergraduate medical students, i.e. the four-year program, the five-year program and the eight-year program. The four-year program covers students majoring in bioscience, biomedical engineering, medical examination, rehabilitation therapy, nursing, bioinformatics, health service administration and biotechnology. The five-year program covers students majoring in clinical medicine, basic medical science, anesthesiology, medical imaging, ophthalmology, psychiatry, pediatrics, dentistry, preventive medicine, pharmacy and forensic medicine. The eight-year program covers students majoring in clinical medicine and students in the eight-year program do not take entrance exams for postgraduate study and they can get a Master’s Degree or a Doctor’s Degree in Medicine after they finish 8 years of study. Considering the course schedule and teaching resources, our institution made the decision that as a pilot program, CSMHEC was given to some first-year medical students in the four-year program and in the five-year program, who are majored in preventive medicine, nursing, medical imaging, biomedical engineering, basic medical science and health service administration.

### Study design

**Fig. 1:**
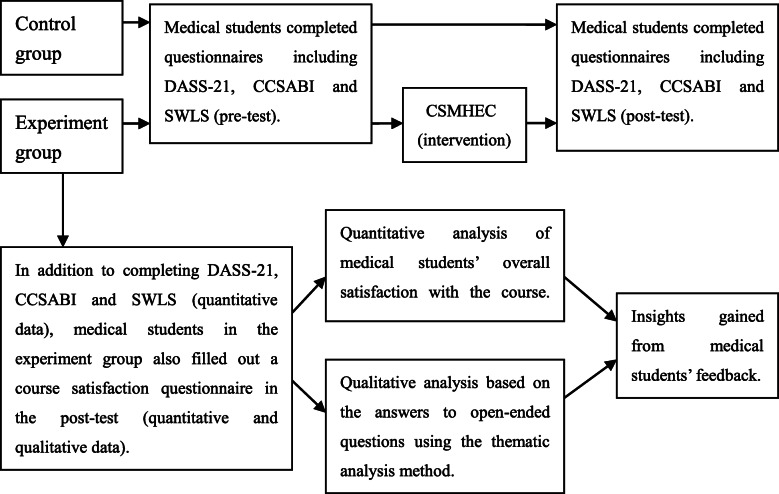
Design of the current research

This is a quasi-experimental study with both quantitative and qualitative analyses. For the quantitative analysis, a quasi-experiment was conducted to test the effectiveness of the intervention (CSMHEC). The measurement tools of medical students’ mental health included Depression Anxiety Stress Scales-21 (DASS-21), Chinese College Student Academic Burnout Inventory (CCSABI) and the Satisfaction with Life Scale (SWLS). In the first course of CSMHEC, students in the experiment group were asked to finish these psychological scales (pre-test) and in the last course of CSMHEC, besides completing the psychological scales again (post-test), students were also invited to fill out a questionnaire about their satisfaction with CSMHEC and their opinions about the course. This course satisfaction questionnaire resulted in quantitative data from responses to a Likert-type question, and qualitative data from textual comment responses to open-ended questions. For the qualitative data, we used a thematic analysis method to gain some insights on how the course could be improved from the implementation of CSMHEC based on medical students’ feedback. The students in the control group completed the same kind of psychological scales at about the same time as the students in the experiment group, but students in the control group did not take CSMHEC and thus did not fill out the course satisfaction questionnaire. The design of the current research is presented in Fig. 1.

### Participants and data collection

In the last week of September 2019, the research investigator from the department of psychology (the corresponding author of this paper and also one of the lecturers of CSMHEC) explained the purpose of the study to students in the first course of CSMHEC to recruit subjects for the experiment group. At approximately the same time in the same week, trained research investigators from the English department tried to help recruit medical students who did not take CSMHEC as subjects for the control group. We used random cluster sampling method, and 374 first-year medical students agreed to participate and became our subjects. As for the demographic characteristics, there were 188 medical students in the experiment group (aged 17.97 ± 0.65 years, 37.2% male) and 186 students in the control group (aged 18.02 ± 0.63 years, 40.3% male). Student’s t-test showed that there was no significant difference in the two groups in terms of medical student age (t = − 0.730, *p* > 0.05). The result of Chi-square test revealed that there was no significant difference in the two groups with regard to gender (*χ*^*2*^ = 0.376, *p* > 0.05). Participants did not know which group they belonged to and research investigators received the training for conducting the survey beforehand. Data were collected through an online survey tool called Star Questionnaire. First, the purpose of the survey (i.e. “for research only”) was explained by each trained research investigator in person in class and then a two-dimensional code was projected onto a large screen. Participants scanned the two-dimensional code using their cellular phones and filled out the questionnaires online.

### Ethical approval

This study was approved by the Institutional Review Board of the authors’ affiliated university and complied with the code of the Declaration of Helsinki (1964) and its later amendments. Medical students were assured that participation was voluntary and they could withdraw anytime if they wanted to without any punishment. Students were told that all data obtained from the survey would be kept confidential and would be used for research purposes only. Every medical student who agreed to participate filled out questionnaires and the informed consent form online.

### Measures

#### Psychological distress

Medical students’ psychological distress was measured by the Depression Anxiety Stress Scales-21 (DASS-21) [17]. This 21-item questionnaire contained three subscales: Depression (7 items) (e.g. “I could see nothing in the future to be hopeful about”); Anxiety (7 items) (e.g. “I was worried about situations in which I might panic and make a fool of myself”); Stress (7 items) (e.g. “I tended to over-react to situations”). Students were asked about their emotional state in the past week and each item was rated on a 4-point Likert scale ranging from 1 (does not apply to me at all) to 4 (apply to me very much), with higher scores indicating higher levels of psychological distress. DASS-21 has been used in Chinese university students and demonstrated good psychometric properties [18]. In the current study, the Cronbach’s alpha coefficients of the scale for the experiment group were 0.895 (pre-test) and 0.922 (post-test) and the Cronbach’s alpha coefficients of the scale for the control group were 0.909 (pre-test) and 0.933 (post-test).

#### Academic burnout

Medical students’ overall levels of academic burnout were measured by the Chinese College Student Academic Burnout Inventory (CCSABI) [19]. Based on Maslach Burnout Inventory, Lian and colleagues developed CCSABI considering Chinese college students’ characteristics and demonstrated the sound psychometric properties of the questionnaire. CCSABI included 20 items and each item was rated on a 5-point Likert scale from 1 (not true of me at all) to 5 (very true of me). Example questions in CCSABI are “When I wake up in the morning, thinking about studying for the whole day, I feel exhausted” and “I can catch up with my academic work in college”. After the negatively worded items were reverse scored, the sum score was calculated with higher sum scores indicating higher levels of academic burnout. CCSABI is now the most widely used academic burnout inventory among college students in China and demonstrated good reliability among Chinese medical students [20, 21]. The Cronbach’s alpha coefficients of CCSABI for the experiment group in the present study were 0.804 (pre-test) and 0.885 (post-test) and the Cronbach’s alpha coefficients for the control group were 0.819 (pre-test) and 0.890 (post-test).

#### Life satisfaction

Life satisfaction, as a major component of subjective well-being, was measured with the 5-item Satisfaction With Life Scale (SWLS) [22]. Example items of SWLS are “In most ways, my life is close to my ideal” and “So far, I have gotten the important things I want in life”. Medical students rated each item on a 7-point Likert scale and the total score ranges from 7 to 35 with a higher score indicating a higher level of life satisfaction. The Chinese version of SWLS has been used in medical students and has demonstrated satisfactory reliability [23]. In the present study, the Cronbach’s alpha coefficients of the scale for the experiment group were 0.865 (pre-test) and 0.903 (post-test) and the Cronbach’s alpha coefficients for the control group were 0.835 (pre-test) and 0.893 (post-test).

#### College student mental health education course (CSMHEC)

In the last week of September 2019, participants in the experiment group took an intensive 8-session mental health education course, the CSMHEC. The course took 2 months (8 weeks), 1.5 h per session and one session per week, so altogether there were 12 h of theoretical lectures. The lectures were given by four specialists from the department of psychology in China Medical University and they all held Master’s Degrees in Psychology. The content and aims of CSMHEC are presented in Table 1.

**Table 1:** The course content and aims of CSMHEC

Modules	Teaching Content (for teachers)	Aims and Goals (for students)
1. General Theory of Mental Health (1.5 h)	·Basic concepts in psychology ·Psychological processes ·Common psychological problems among college students ·Factors affecting college students’ mental health	·To understand basic concepts in psychology ·To acquire knowledge about psychological processes ·To be familiar with common mental health problems and the associated factors among college students
2. Knowing Oneself: the Development of Self-awareness (1.5 h)	·The concept of self-awareness ·The development of self-awareness (egocentric stage, objectification, subjectization) ·Deviation and psychological adjustment in the development of college students’ self-awareness	·To understand the meaning of self-awareness and the different stages in the development of self-awareness ·To get familiar with the characteristics of college students’ self-awareness development ·To grasp the approaches to forming a healthy level of self-awareness
3. Personality Building (1.5 h)	·The meaning and characteristics of personality ·The relationship between personality and mental health ·Personality defects and correction	·To grasp the meaning and characteristics of personality ·To understand the relationship between personality and mental health ·To be familiar with the common personality defects of college students and corrective measures
4. Psychological Adjustment in the Learning Process (2 sessions) (1st session 1.5 h)	·The definition of learning motivation ·How to stimulate learning motivation ·Methods to develop learning potential	·To understand the learning characteristics and psychological mechanism of college students ·To activate learning motivation ·To cultivate learning ability and develop learning potential
5. Psychological Adjustment in the Learning Process (2nd session 1.5 h)	·Basic characteristics of college students’ learning activities ·Common learning barriers among college students (conflict of motives, cognitive impairment, attention disorder, test anxiety, learning fatigue) ·Psychological adjustment methods	·To be familiar with basic characteristics of college students’ learning activities ·To understand the common learning barriers of college students ·To grasp the psychological adjustment methods and overcome learning barriers
6. Emotion Management (2 sessions) (1st session 1.5 h)	·The concept and characteristics of emotions ·College students’ emotional characteristics and the influences of emotions on mental health, cognition and behaviors ·Cultivate positive emotions	·To understand the concept and characteristics of emotions ·To be familiar with characteristics of college students’ emotions and their influences ·To develop a positive attitude
7. Emotion Management (2nd session 1.5 h)	·Common college students’ negative emotions ·Depression and the adjustment (rational emotion therapy, five steps out of depression, venting exercise therapy) ·Anxiety and the adjustment (accept your emotions, shift your focus, cognitive therapy)	• To be familiar with common negative emotions among college students • To understand the concept of depression and grasp the adjustment method • To understand the concept of anxiety and grasp the adjustment method
8. Coping with Stress (1.5 h)	·The concept of stress ·Major causes of stress ·Stress coping strategies (capacity building, social support, behavioral regulation, psychological adjustment)	• To be familiar with the concept of stress and understand the major causes of stress • To master the stress coping strategies in order to combat the stress-related mental health problems

Note. CSMHEC also contains some content on interpersonal skills in the two-session Modules 4–5 and Modules 6–7, please refer to the Additional file1

### Analyses

#### Quantitative analysis

Independent samples t-tests were used to compare baseline levels of psychological study variables (DASS-21, CCSABI, SWLS) between the experiment group and the control group and to compare levels of psychological study variables between the experiment group and the control group after the intervention. Paired samples t-tests were used to compare the levels of psychological study variables in the experiment group before and after the students in the group took CSMHEC and were used to compare the levels of psychological study variables in the control group in the pre-test and post-test without the intervention. All t-tests were performed by SPSS 22.0 version and a *p* value of less than 0.05 was considered statistically significant. For paired samples t-tests and independent samples t-tests, effect size was measured by Cohen’s *d* using GPower 3.1. According to Cohen [24], effect size was interpreted as small (Cohen’s *d ≥* 0.20), medium (Cohen’s *d ≥* 0.50) and large (Cohen’s *d ≥* 0.80). As for medical students’ feedback about the course CSMHEC in the experiment group, SPSS 22.0 was employed to analyze the quantitative data about students’ overall satisfaction with CSMHEC.

#### Qualitative analysis

Thematic analysis method was adopted to gain insights from medical students’ answers to open-ended questions (qualitative data) in the feedback. The open-ended questions used in the course satisfaction questionnaire were designed by QHW (the first author) and then QHW discussed with TJD (the corresponding author). Considering the limited time issue in class, after discussion, QHW and TJD finally reached the agreement that the number of open-ended questions asked in the survey was reduced from five to three. Students in the experiment group typed in their answers to the three open-ended questions using their cellular phones through an online survey tool called Star Questionnaire and then the texts were exported. In order to elicit honest answers from students and to control bias, the course satisfaction questionnaire was answered in complete anonymity without any identifier. For qualitative analysis, based on principles of grounded theory [25], an inductive thematic analysis approach [26] was adopted in the current study. First, QHW encoded the exported texts and extracted the concurrent words to search for themes. Then a thematic ‘map’ was generated and a discussion was held between QHW and TJD. Themes extracted initially were compared and further refined and the finalized themes were agreed upon by both authors after a consensus was reached.

## Results

### Comparisons of baseline levels of psychological variables between the experiment group and the control group

**Fig. 2:**
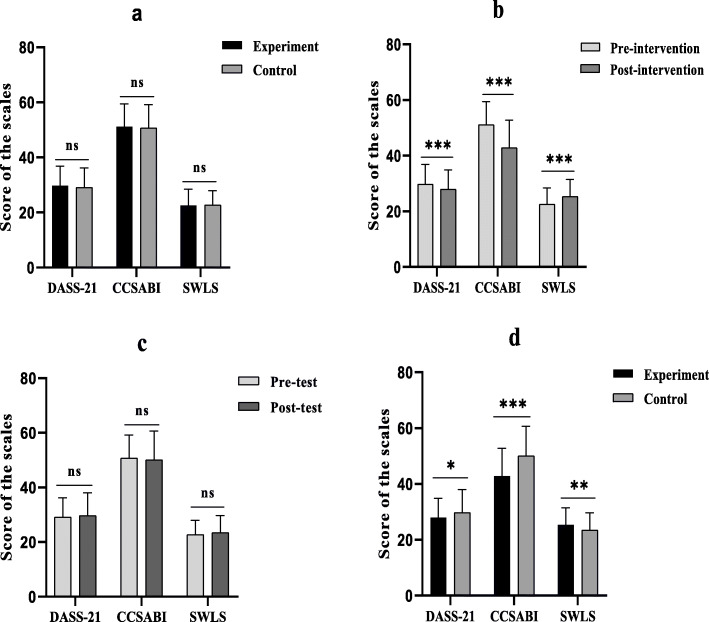
Results of the quasi-experiment. **a.** Comparisons of baseline levels of psychological variables between the experiment group and the control group. **b.** Comparisons of psychological variables in the experiment group before and after the intervention. **c.** Comparisons of psychological variables in the control group between the pre-test and the post-test without the intervention. **d.** Comparisons of psychological variables between the experiment group and the control group in the post-test. (Note. the experiment group: *N* = 188; the control group: *N* = 186; DASS-21: the 21-item Depression, Anxiety and Stress Scales; CCSABI: Chinese College Student Academic Burnout Inventory; SWLS: Satisfaction With Life Scale; ns: not significant; *p < 0.05; **p < 0.01; ***p < 0.001)

Independent samples t-tests were used to compare the psychological variables between the experiment group and the control group before implementing CSMHEC in the experiment group. The results presented in Fig. 2a show that there were no significant differences between the experiment group and the control group regarding medical students’ levels of psychological distress (29.74 ± 7.09 vs 29.08 ± 7.06, t = 0.915, *p* > 0.05), academic burnout (51.16 ± 8.26 vs 50.73 ± 8.42, t = 0.503, *p* > 0.05) and life satisfaction (22.56 ± 5.87 vs 22.68 ± 5.29, t = − 0.215, *p* > 0.05). The homogeneity of the two groups in baseline levels of the psychological study variables laid the foundation for testing the effectiveness of the intervention.

### Comparisons of psychological variables in the experiment group before and after the intervention

Figure 2b shows the levels of psychological variables in the experiment group before and after medical students took CSMHEC, which were compared by paired samples t-tests. The results reveal that after taking CSMHEC, students in the experiment group had significantly lower levels of psychological distress (29.74 ± 7.09 vs 27.97 ± 6.92, t = 4.244, *p* < 0.001, *d* = 0.31) and significantly lower levels of academic burnout (51.16 ± 8.26 vs 42.85 ± 9.94, t = 19.997, p < 0.001, *d* = 1.46). Meanwhile, Medical students’ life satisfaction levels were significantly higher (22.56 ± 5.87 vs 25.35 ± 6.09, t = − 6.588, *p* < 0.001, *d* = 0.48) after the intervention.

### Comparisons of psychological variables in the control group between the pre-test and the post-test without the intervention

Medical students in the control group completed the same psychological scales at approximately the same time as those in the experiment group. Without taking CSMHEC, medical students in the control group finished the same questionnaires twice in the pre-test and in the post-test with 2 months interval and the results of paired samples t-tests are presented in Fig. 2c. As can be seen, medical students in the control group saw no significant differences in their levels of psychological distress (29.08 ± 7.06 vs 29.73 ± 8.27, t = − 1.117, *p* > 0.05), academic burnout (50.73 ± 8.42 vs 50.08 ± 10.64, t = 1.114, *p* > 0.05), or life satisfaction (22.68 ± 5.29 vs 23.45 ± 6.22, t = − 1.684, *p* > 0.05) between the pre-test and the post-test.

### Comparisons of psychological variables between the experiment group and the control group after implementing CSMHEC

To compare medical students’ levels of the studied psychological variables between the experiment group and the control group after the intervention, independent samples t-tests were used. Figure 2d shows that after taking CSMHEC, students in the experiment group had a significantly lower level of psychological distress than those in the control group (27.97 ± 6.92 vs 29.73 ± 8.27, t = − 2.230, *p* < 0.05, *d* = 0.23). It is noticeable that the academic burnout level of the students in the experiment group was also significantly lower than that of the students in the control group with a larger effect size (42.85 ± 9.94 vs 50.08 ± 10.64, t = − 6.792, *p* < 0.001, *d* = 0.70). On the other hand, students in the experiment group saw a significantly higher level of life satisfaction than students in the control group (25.35 ± 6.09 vs 23.45 ± 6.22, t = 2.983, *p* < 0.01, *d* = 0.31).

### Medical students’ overall satisfaction with CSMHEC

**Fig. 3:**
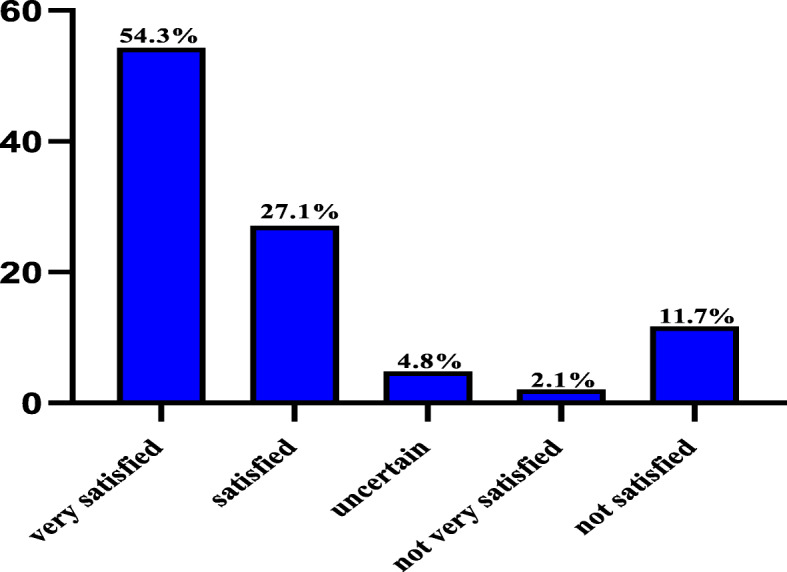
Survey about medical students’ overall satisfaction with CSMHEC

In the last course of CSMHEC, medical students in the experiment group (*N* = 188) completed a course satisfaction questionnaire and the result of analysis of quantitative data is shown in Fig. 3. As can be seen from the bar chart, a large majority of students were either very satisfied with the course (*n* = 102, 54.3%) or satisfied with the course (*n* = 51, 27.1%). Students who were unsure about their attitudes towards the course accounted for a small proportion (*n* = 9, 4.8%). It is noticeable that four students were not very satisfied with the course (2.1%) and the percentage of medical students who were not satisfied with the course at all was much larger (*n* = 22, 11.7%).

### Thematic analysis of medical students’ qualitative feedback on CSMHEC

Through asking open-ended questions, we collected feedback from medical students in the experiment group after they took CSMHEC and analyzed the qualitative data using the thematic analysis method. Details about the open-ended questions, the extracted themes and students’ quotes are presented in Table 2. As can be seen from the table, most students endorsed the value of the course in helping them to manage their emotions more effectively, familiarizing them with basic concepts in psychology, having a clearer idea of themselves and getting to know the common mental health problems among college students and strategies to cope with these problems. Regarding the good aspects of the course, a large number of students claimed that when taking CSMHEC, they felt relaxed and interested, the course content was closely related to their real life in college and they thought that the course lecturers were kind and responsible. As for the aspects of the course that need to be improved, students presented the view that they preferred more interaction, more cases and more E-educational activities.

**Table 2:** Thematic analysis of the feedback on CSMHEC from medical students in the experiment group (*N* = 188)

Open-ended questions	Themes and Quotes (n/N)
**What’s your biggest gain from taking CSMHEC?**	**Helpful in emotion management (63/188)** “…*know how to control my feelings effectively”* “*By taking this course, I learn to manage my emotions.”* “*I have a better understanding of the changes in my feelings and can adjust myself.”* “*When I feel sad, I know I need to vent and do not let the feeling disturb me…”* **Familiar with basic theories in Psychology (47/188)** “*I learn more theories about psychology”* “*...helped me understand some concepts in psychology…”* “*...get some knowledge about mental health”* “*After taking CSMHEC, I get to know the definition of psychology and the importance of mental health…”* **Have a clearer idea about the self (28/188)** “*CSMHEC makes me understand myself better…”* “*I know myself better and I need to keep a positive attitude towards life.”* “*…have a deeper understanding about my mental state.”* “*I know what my problems are… I should not ruminate...”* **Know common mental health problems among college students and strategies to cope with them (19/188)** “*I’m familiar with the symptoms of depression and how to deal with it.”* “*…understand the features of college students’ psychological development…”* “*I know how to relax and get the strategies to cope with stress.”* “*After taking CSMHEC, I get an idea about mental health problems that college students may have and what to do.”*
**Which aspects of CSMHEC do you think are good?**	**Learning in a relaxing atmosphere (56/188)** “*When I am taking CSMHEC, I feel relaxed.”* “*It is relaxing in class and I gain some knowledge on psychology”* “*In a relaxing classroom environment, we can communicate with the teacher and classmates well…”* **Interesting, vivid and informative (51/188)** “*I think this course is interesting and I can learn a lot.”* “*…some examples are interesting and vivid, I’m eager to learn”* “*Good classroom atmosphere…I do not feel bored…get a lot of knowledge on mental health…”* **Teachers are kind and patient, giving detailed explanations (35/188)** “*…different modules are taught by different teachers, they are all very nice…”* “*When explaining a difficult point, the teacher is very patient and set some examples for us…”* “*Some theories are hard to understand. However, teacher will explain them in simple language…”* **Close to college students’ real life and very practical (23/188)** “*The cases in the book are about college students’ mental health problems and are very useful for us.”* “*…examples are close to our real life in college…”* “*…related to our life…real and practical…”*
**Which aspects of CSMHEC do you think need to be improved?**	**More interaction suggested (44/188)** “*I think teachers need to have more interaction with us.”* “*It is better to have more communication between the teacher and us, between our students…”* “*I want to interact with teachers and classmates more.”* “*…more teamwork and discussion…* *“…more interesting interactive activities can be added…”* **More cases preferred (37/188)** *“Some cases about college students’ mental health are very interesting. I hope there are more such cases.”* *“…provide more cases which are close to college students’ life…”* *“Teachers can play more videos about the cases and then we can discuss...”* *“I think it would be more interesting to have more cases to analyze.”* *“For the course content, I want to see more cases…that are practical…”* **More E-education favored (32/188)** *“Teachers can use more online software to send us a survey to do, and this will attract more students to actively participate in class activities.”* *“I think the electronic interaction is convenient…teachers set some questions and we answer them online, in this way, we can keep our attention on the course content, that’s good…”* *“Using cell phones to do a survey online is very convenient, stimulate us to participate…”* *“I think if the teacher can give us some award-winning quiz using online software, it would be interesting and attract more students to participate…”*

## Discussion

This quasi-experimental study tested the effectiveness of implementing an intensive 8-week mental health education course in undergraduate medical curriculum and tried to gain insights from students’ feedback on how the course could be improved. Statistics show that after taking CSMHEC, medical students in the experiment group saw a significant decline in psychological distress and academic burnout, while their levels of life satisfaction increased significantly. Compared with students in the control group, students in the experiment group had significantly lower levels of psychological distress and academic burnout but significantly higher levels of life satisfaction in the post-test. This result is encouraging and confirmed that at least the short-term effect of CSMHEC was significant. Three themes, with interaction, case and E-education as the key words, emerged from qualitative data in the thematic analysis, giving further insights for the aspects of the course that need to be improved.

### Embedding mental health education course early in medical curriculum

The College Student Mental Health Education Course, as a pilot program, is not a compulsory course for all first-year students at the medical university where the present study was conducted. In China, students get admitted to medical universities directly after finishing their study at senior high schools through Chinese National College Entrance Exam, so it is likely that many students are not clear of the challenges they will have to face in the process of learning medicine. Literature shows that mental health problems such as depression, anxiety and burnout may set in from the outset of medical education [27, 28]. The first academic year in medical university is a crucial transitional period for medical students in China and differences in the learning environment and teaching methods between senior high schools and medical universities may act as stressors. Some medical students cannot adapt to the new academic environment and teaching mode, thus feeling stressed and suffering from mental health problems like anxiety and burnout. Therefore, early intervention is needed and implementing a mental health education course like CSMHEC in medical curriculum may be a promising approach.

CSMHEC helped medical students understand the features of college students’ psychological development and the related psychological problems that may arise in this process. Through taking CSMHEC, medical students could know basic theories in psychology, develop a healthy level of self-awareness, get familiar with common mental health problems (stress, anxiety, depression, burnout) they may encounter in the process of learning medicine and grasp the coping strategies to combat these problems. As a general education course, CSMHEC was given to whole classes of medical students, so students taking the mental health education course did not have to worry about stigma. Literature shows that medical students and physicians had lower levels of help-seeking and were reluctant to use public psychiatric services when they suffered from mental health problems due to stigmatization [29–31]. It was expected that CSMHEC could not only increase medical students’ mental health literacy, but also break their moulds of thinking and enable them to realize some inherent unreasonable perceptions, thereby changing their maladaptive behaviors. It is hoped that after taking the course, in the future, if the students were afflicted by mental illnesses, they could actively seek assistance from the counseling center on campus or from public mental health services.

### Insights on how the course could be improved

The teaching of CSMHEC was still based on didactic lectures, combined with some interaction mainly through questions asked by teachers in class. Sometimes medical students also interacted with each other through small group discussions on a course-related topic or around a specific case assigned by teachers. Medical students in the experiment group presented the view that they preferred more interaction with teachers and peers. Previous research has revealed that interactive education is an effective teaching mode that can take different forms in medical education [32–34]. It is suggested that medical educators pay more attention to teacher-student and student-student communication in class.

At the end of each module of CSMHEC, there were several cases about college students’ psychological problems related to the content of that specific module. The purpose was to nurture medical students’ critical thinking, analytical ability and problem-solving skills. First, medical students needed to understand the problems presented in the cases, and then reflected upon the knowledge they acquired in the related module so as to figure out the appropriate approaches to tackling the problems. Through this process, it is intended that medical students could internalize the knowledge acquired and more importantly, could integrate theory with practice. Students in the experiment group said that they thought some cases were interesting which aroused their curiosity and eagerness to learn. Extant literature has recorded the effectiveness of the case-based approach in medical education [35, 36] and medical educators can make the most of this approach to facilitate their teaching.

With the advance of technology and easy access to the Internet, the teaching and learning modes are undergoing dramatic changes. Various online educational softwares such as Yun Class, TengXun Class and U Class are now very popular in China. Medical teachers can make good use of such E-educational software to supplement the traditional blackboard and PowerPoint teaching format. For example, in the preparation stage before class, medical teachers can set different forms of questions (multiple choice questions, filling in the blanks, etc.) or make a survey specifically related to the content of the course. During class hours, after didactic teaching of some theories, medical teachers can ask students to answer the related questions or fill out the survey online using their cellular phones. A great advantage of this approach is that E-educational software may calculate the correct rate for each question immediately and with reference to the statistics, medical teachers are able to identify medical students’ knowledge gaps and make demonstrations accordingly. In the present study, we also used an online survey tool called Star Questionnaire for collecting the quantitative and qualitative data. Medical students taking CSMHEC in the experiment group said that they welcomed this teaching format of combining the traditional didactic lecture with the modern electronic interaction and they preferred more utilization of the E-educational software. Empirical studies have demonstrated the effectiveness of using online teaching resources in medical education [37, 38] and medical educators can combine the traditional face-to-face didactic lectures with modern on-line interactions to make their teaching more effective.

### Strengths and contributions of the current research

Although a large number of cross-sectional studies reported medical students’ mental health problems and different psychotherapeutic programs have been developed to help medical students improve psychological well-being, we found that there are few studies focusing on the effectiveness of a mental health education course to this end. Zhang et al. [39] adopted a pre-post intervention design and applied a positive psychology education course for medical students in China. Results show that after the intervention, participants’ levels of hope, life satisfaction and subjective happiness were improved significantly, while their levels of depression and anxiety were reduced considerably. Compared with Zhang’s study, our research used a larger sample of medical students and adopted a quasi-experimental design with a control group to test the effectiveness of implementing a mental health education course in medical curriculum, thus adding testing power to the result of the trial. In addition, our research also included qualitative analysis which provided further insights on the better design of such a mental health education course to implement in medical education. Our research, together with Zhang’s study, provided evidence that embedding a classroom-based mental health education course in regular medical curriculum can be a feasible and effective approach to helping medical students enhance psychological health.

### Limitations and future directions

Whereas the present study contributes to the literature on developing effective intervention programs to help improve medical students’ psychological health, we have to acknowledge that our study has several limitations. First, we could not use an RCT design but a quasi-experimental design instead because our institution arranged to give CSMHEC to some but not all first-year medical students due to their different course schedules. Second, students in the experiment group may have the expectations that their psychological well-being would improve after taking the mental health education course which could amplify the better outcomes in the intervention group. Third, this study was conducted in only one medical university in China, so generalizability of the conclusions should be made with caution. Fourth, we did not do follow-up tests for medical students in the experiment group due to the COVID-19 pandemic outbreak as we thought this traumatic event would have exerted considerable impacts on the mental state of our subjects. Considering these limitations, future studies in different cultures need to be conducted, if possible, by using an RCT design and with follow-up tests.

## Conclusions

This study tested the efficacy of implementing an intensive 8-week mental health education course CSMHEC in medical education. The results demonstrated the effectiveness of the course on decreasing medical students’ psychological distress and academic burnout while enhancing their life satisfaction levels and students’ qualitative feedback shed light on how the course could be improved. Medical institutions and the related authorities may consider implementing a mental health education course like CSMHEC in regular medical curriculum to help medical students improve psychological health.

## Supplementary Information

**Additional file 1.** Content on interpersonal skills integrated into CSMHEC.

## Data Availability

The datasets used and/or analyzed in the present study are available from the corresponding author on reasonable request.

## Abbreviations

CSMHEC: College Student Mental Health Education Course
DASS-21: The 21-item Depression Anxiety Stress Scales
CCSABI: Chinese College Student Academic Burnout Inventory
SWLS: Satisfaction With Life Scale
MHFA: Mental Health First Aid
RCT: Randomized controlled trial
PMR: Progressive Muscle Relaxation
CBT: Cognitive behavioral therapy
