# Supplementary material for: Implementation of the college student mental health education course (CSMHEC) in undergraduate medical curriculum: effects and insights
Source: BMC Med Educ. 2020 Dec 11;20:505. doi: 10.1186/s12909-020-02438-1 (PMC7731140; doi:10.1186/s12909-020-02438-1)
Supplement: Supplementary file 1 — Additional file 1. Content on interpersonal skills integrated into CSMHEC. [file 12909_2020_2438_MOESM1_ESM.doc]

**Supplementary Table1**

Content on psychological adjustment in interpersonal relationships integrated in College Student Mental Health Education Course (CSMHEC)

| Improving social skills | ·Factors influencing interpersonal relationships  ·Psychological effect in interpersonal relations  ·Methods and skills in building harmonious interpersonal relationships (e.g. with teachers, classmates, dorm mates, etc.) |
| --- | --- |
| Psychological adjustment  in romantic relationships | ·Psychological development of love in college students  ·Psychological characteristics among college student lovers  ·Psychological confusion and emotion regulation in romantic relationships |
